# Nitrogen-Doped Biochar Aerogel as Efficient Peroxymonosulfate Activator for Organic Pollutant Removal

**DOI:** 10.3390/nano15110865

**Published:** 2025-06-04

**Authors:** Lingshuai Kong, Mingshuo Zhu, Jinhua Zhan

**Affiliations:** 1 Institute of Eco-Environmental Forensics, School of Environmental Science and Engineering, Shandong University, Qingdao 266237, China; 2 Key Laboratory of Colloid and Interface Chemistry, Ministry of Education, School of Chemistry and Chemical Engineering, Shandong University, Jinan 250100, China; zhumingshuo1996@163.com; 3 Shandong Coal Science and Technology Research Institute Branch, Yankuang Energy Group Co., Ltd., Jinan 250014, China

**Keywords:** peroxymonosulfate, biochar, aerogel, organic pollutant

## Abstract

Rapid industrialization has escalated environmental pollution caused by organic compounds, posing critical challenges for wastewater treatment. Advanced oxidation processes based on peroxymonosulfate (PMS) suffer from metal leaching and catalyst recycling challenges. To address these limitations, this study developed a nitrogen-doped biochar aerogel (NBA) derived from poplar wood powder as an eco-friendly and easily recoverable PMS activator. The NBA catalyst, optimized by tuning the calcination temperature to achieve a specific surface area of 297.5 m^2^ g^−1^, achieved 97% bisphenol A (BPA) removal within 60 min with a catalyst dosage of 0.3 g/L and 1.0 mM PMS under mild conditions. The material exhibited broad pH adaptability (pH 3.5–9), recyclability (>94% efficiency after thermal treatment), and versatility in degrading seven pollutants (BPA, phenol, 4-chlorophenol, 2,4-dichlorophenol, 2,4,6-trichlorophenol, rhodamine 6G, and levofloxacin) through synergistic radical (•OH, SO_4_^•−^, O_2_^•−^) and non-radical (^1^O_2_) pathways. X-ray photoelectron spectroscopy (XPS) analyses revealed that nitrogen doping enhanced PMS activation by optimizing electronic structures. This study highlights the potential of waste biomass-derived carbon aerogels as eco-friendly, efficient, and reusable catalysts for advanced oxidation processes in wastewater treatment.

## 1. Introduction

With the rapid development of industrialization, numerous types of industrial organic compounds enter the environment, leading to the pollution of aquatic ecosystems [1]. The treatment of refractory organic compounds is one of the most critical and challenging problems in the environmental remediation process [2]. Advanced oxidation processes (AOPs), which involve the in situ generation of highly reactive species, are widely used for the degradation of refractory organic compounds in industrial wastewater and drinking water [3]. As one of the prominent oxidants in AOPs, peroxymonosulfate (PMS, HSO_5_^−^) with an asymmetric structure can be activated by metal-based and metal-free materials to generate multiple reactive species, including sulfate radical (SO_4_^•−^), hydroxyl radical (^•^OH), superoxide radical (O_2_^•−^), singlet oxygen (^1^O_2_), catalyst-PMS complexes, among others [4,5,6,7]. AOPs based on PMS have been successfully applied to degrade various pollutants. However, there are still inherent drawbacks in practical applications, such as (i) short-lived radical species limiting mass transfer [8,9]; (ii) challenges in recycling powder catalysts; and (iii) secondary pollution caused by metal ion leaching [10,11].

Transition metal activation of PMS is highly effective in degrading organic pollutants [12,13]. However, inevitable metal leaching increases environmental risks, hindering the practical application of transition metal catalysts in environmental remediation. Compared to traditional metal-based catalysts such as iron and cobalt, carbon catalysts offer advantages, including environmental friendliness, corrosion resistance, and biocompatibility, while overcoming issues like sintering and metal leaching inherent to metal-based systems [14,15,16]. Despite these benefits, pure carbon materials exhibit limited activation ability. Consequently, heteroatom-doped carbon nanoparticles have garnered significant attention. Nitrogen doping, for instance, enhances PMS activation by modifying spin and charge distribution in carbon matrices [17]. Nitrogen-doped graphene, carbon nanotubes, and graphene–biochar aerogels have proven effective as PMS activators for pollutant degradation [17,18,19]. However, conventional carbon sources like graphene oxide (GO) or carbon nanotubes face limitations in scalability, cost, and sustainability. In contrast, biochar derived from waste biomass provides a low-cost, renewable, and easily processable alternative, making it an ideal precursor for carbon catalyst production [20,21].

The rapid global increase in solid waste generation has become one of the most pressing environmental challenges. As a renewable biomass material, poplar wood powder has attracted increasing attention as a cost-effective, widely available, and sustainable resource. Rich in cellulose and lignin, it offers excellent carbonization properties and a natural porous structure, making it an ideal precursor for the fabrication of functional biochar materials [22,23]. Additionally, studies indicate that wood powder can be utilized as a raw material to produce biochar aerogels. Biochar aerogels are unique porous materials consisting of interlinked carbon nanomaterials and interstitial mesopores (2–50 nm) [24]. This monolithic structure offers a high specific surface area, facile recyclability, and excellent mechanical performance, making it particularly advantageous for adsorption, filtration, and catalytic applications compared to traditional powder catalysts [25,26].

In this study, nitrogen-doped biochar aerogel (NBA) was prepared from poplar wood powder derived from forest waste and used as an activator for PMS. The prepared NBA catalyst was systematically characterized, and its ability to activate PMS for bisphenol A (BPA) degradation was tested. Meanwhile, optimal calcination conditions were determined by adjusting the calcination temperature. Key factors affecting BPA degradation were investigated, and the capability of NBA-activated PMS in pollutant degradation was further evaluated in terms of universality and reusability. Finally, the BPA degradation mechanism was elucidated, with potential reactive sites proposed.

## 2. Materials and Methods

### 2.1. Chemicals

Sodium hydroxide (NaOH), hydrogen peroxide (H_2_O_2_, ≥30.0%), sulfuric acid (H_2_SO_4_), p-chlorophenol (4-CP), rhodamine 6G (Rh6G), tert-butyl alcohol (TBA), 2,4-dichlorophenol (2,4-DCP), 2,4,6-trichlorophenol (2,4,6-TCP), levofloxacin, urea, and phenol were obtained from Sinopharm Chemical Reagent Co., Ltd. (Shanghai, China). Peroxymonosulfate (2KHSO_5_·KHSO_4_·K_2_SO_4_, PMS, ≥47.0% KHSO_5_ basis), bisphenol A (BPA), furfuryl alcohol (FFA), superoxide dismutase (SOD, ≥1400 units/mg) were purchased from Aladdin Chemical Co., Ltd. (Shanghai, China). Methanol and ethanol were obtained from Tianjin Fu Yu Fine Chemicals Co., Ltd. (Tianjin, China). All the chemicals were used without any further purification. Deionized water was used to prepare all aqueous solutions (18.25 MΩ·cm). Poplar sawdust was obtained from a wood processing factory in Jinan, Shandong, China, where it was generated as waste material from the mechanical cutting of poplar wood during board manufacturing. The resulting powder was then ground and sieved through a 120-mesh sieve prior to experimental use.

### 2.2. Synthesis of Biochar Catalysts

**Figure 1:**
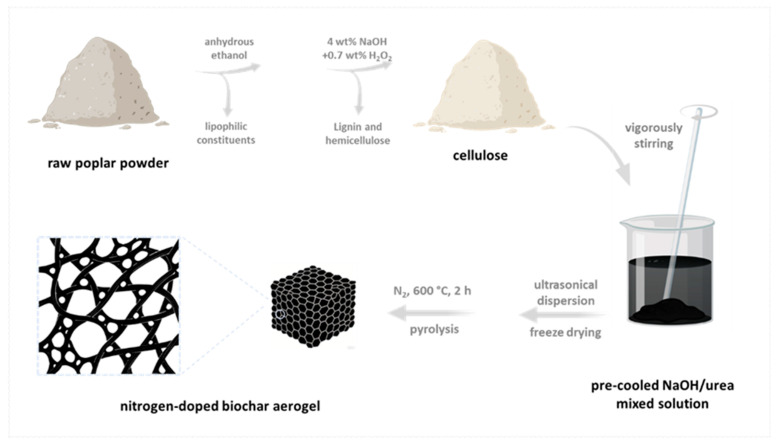
Procedure of the preparation of nitrogen-doped biochar aerogel (NBA).

The nitrogen-enriched biochar aerogel was fabricated from poplar-derived biomass through a multistep purification and carbonization process. Initially, the raw poplar powder underwent solvent dewaxing using anhydrous ethanol to eliminate lipophilic constituents. Subsequently, the delipidated biomass was subjected to alkaline-peroxide treatment in an aqueous solution containing 4 wt% NaOH and 0.7 wt% H_2_O_2_, achieving selective removal of lignin and hemicellulose components while preserving cellulose integrity. Subsequently, the material was immersed in a pre-cooled NaOH/urea mixed solution and vigorously stirred to oxidize cellulose. The reaction solution was ultrasonically dispersed and freeze-dried to form an aerogel. Finally, the aerogel was calcined under a N_2_ atmosphere at 600 °C for 2 h to obtain nitrogen-doped biochar aerogel (labeled NBA, Figure 1). For control samples, dewaxed poplar powder directly calcined under a N_2_ atmosphere at 600 °C was labeled PB. Non-ultrasonicated freeze-dried material calcined under a N_2_ atmosphere at 600 °C was labeled BC.

### 2.3. Catalyst Performance Evaluation

Degradation experiments were conducted in a 100 mL conical flask at 25 °C. In each test series, a predetermined amount of catalyst (15 mg) was dispersed in 50 mL of BPA solution (initial concentration: 10 mg/L) without adjusting the pH. The suspension was subjected to a 20 min dark-phase homogenization to establish thermal equilibrium and adsorption–desorption equilibrium. Then, 1 mL of 20 mM PMS was injected into the conical flask to initiate the reaction. At designated time points, 1 mL samples were extracted, quenched with 0.5 mL ethanol, and rapidly filtered through 0.22 μm PES membranes. Experimental variables, including catalytic loading, PMS concentration, pH conditions (modified using 0.1 M H_2_SO_4_/NaOH), and thermal parameters (25–45 °C), were systematically investigated to optimize degradation efficiency. Post-catalytic NBA was recovered and washed for reusability testing. All pollutant removal experiments were conducted in triplicate to obtain average values. Unless otherwise specified, standard conditions were maintained: [catalyst] = 0.3 g/L, [PMS] = 1 mM, [pollutant] = 10 mg/L, T = 25 °C, with unadjusted pH. The spent catalysts were regenerated through a sequential procedure: soaking in ethanol for 12 h to remove organic residues; repeated washing with deionized water until neutral pH was attained; and calcination at 500 °C for 1 h under a N_2_ atmosphere to restore catalytic activity.

### 2.4. Analytical Methods and Characterization

Concentrations of BPA, phenol, 4-CP, 2,4-DCP, and 2,4,6-TCP were determined by HPLC with C18 reversed-phase chromatography. For BPA, phenol, 4-CP, and 2,4-DCP, the mobile phases were mixed solutions of methanol and water with mixing ratios of 70:30, 80:20, 70:30, and 80:20, respectively. The detection wavelengths were 278 nm, 270 nm, 278 nm, and 284 nm, respectively. The concentration of 2,4,6-trichlorophenol was measured at 290 nm using a mixture of 80% methanol and 20% water (each containing 1% acetic acid) as the mobile phase. The concentrations of levofloxacin and Rh6G were measured at 292 nm and 552 nm using a Puxi UV–Vis spectrophotometer.

The morphology of the catalyst was observed by field emission scanning electron microscopy (FE-SEM, GeminiSEM 300, Carl Zeiss, Jena, Germany). The Brunauer–Emmett–Teller (BET) surface area was obtained by ASAP 2460-4MP (Micromeritics, Norcross, GA, USA) at 77 K, and the pore size distribution was calculated from the corresponding nitrogen absorption and desorption isotherm. The functional groups and surface composition of carbon materials were determined by X-ray photoelectron spectroscopy (XPS, ESCALAB 250Xi, Thermo Fisher Scientific Ltd., Cheshire, UK). The Raman spectrometer (LabRAM HR 800, Horiba/Jobin Yvon, Palaiseau, France) with a 633 nm HeNe laser excitation source was used to analyze the defect structure of the catalyst.

## 3. Results and Discussion

### 3.1. Characterization of Catalysts

**Figure 2:**
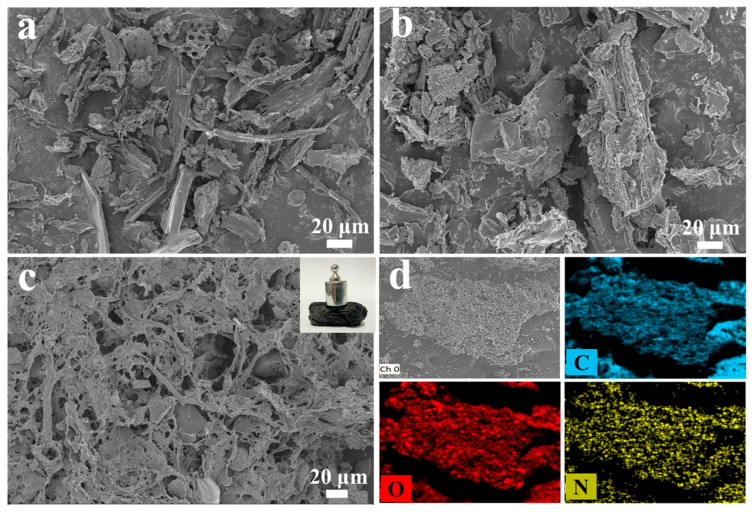
FE-SEM images of (**a**) PB, (**b**) BC, (**c**) NBA, and (**d**) EDS element distribution of NBA.

Under inert conditions, the thermochemical decomposition of lignocellulosic biomass initiates molecular fragmentation through bond cleavage mechanisms. This process facilitates progressive aromatization via cyclization reactions, ultimately resulting in the formation of graphitic carbon structures through solid-phase polycondensation. The resultant carbon-rich material exhibits characteristic features of biochar with enhanced structural ordering [27]. Direct carbonization of dewaxed poplar flour (PB) and purification of poplar flour (BC) resulted in irregular structures (Figure 2a,b). Figure 2c shows the macroscopic morphology and microstructure of NBA, with the inset displaying its macroscopic form. The biochar aerogel exhibits excellent mechanical properties and retains its structural integrity even under mechanical shock. The morphology and structure of the as-prepared samples were characterized using a field emission scanning electron microscope (FE-SEM). Following pyrolysis at 600 °C, the delignified PB demonstrated preservation of its inherent fibrous architecture while undergoing structural fragmentation characterized by the conversion of macroscopic constituents into micron-scale irregular particulates (Figure 2a). Alkali and H_2_O_2_ treatments effectively removed lignin and hemicellulose [28]. The NBA has a porous and interconnected three-dimensional net structure. As shown in Figure 2d, nitrogen (N) is uniformly distributed on the porous nanocarbon skeleton of NBA, confirming the successful preparation of this nitrogen-doped biochar aerogel material.

**Figure 3:**
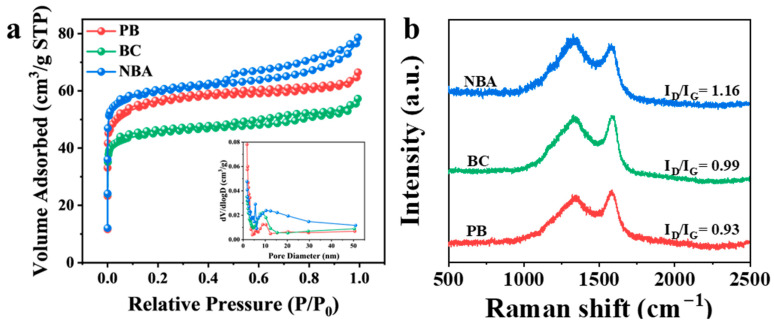
Characterizations of NBA, PB, and BC: (**a**) N_2_ adsorption–desorption isotherms and aperture distribution, (**b**) Raman spectra.

Biochar materials usually have good pore structures. Nitrogen adsorption–desorption analysis was implemented to characterize the BET-specific surface area (SSA) and porous architecture of the catalyst specimens. In accordance with IUPAC nomenclature, all synthesized materials manifested distinct Type IV physisorption isotherm profiles (Figure 3a). The specific surface areas of PB, BC, and NBA were 178.5 m^2^ g^−1^, 142.9 m^2^ g^−1^, and 297.5 m^2^ g^−1^, respectively. Obvious N_2_-type hysteresis loops belonging to the H4 type were observed in NBA, indicating a well-developed hierarchical porous structure [29,30]. NBA showed high N_2_ adsorption capacity at low relative pressure (P/P_0_), suggesting the presence of micropores in NBA. Hysteresis occurred in the range of P/P_0_ = 0.4–1.0, indicating the presence of mesoporous and macroporous. When the relative pressure P/P_0_ > 0.9, the adsorption amount continued to rise, attributed to N_2_ adsorption by macropores [31]. Compared to PB and BC, NBA exhibits advantages in both specific surface area and pore structure. The higher specific surface area and multi-pore structure reduce mass transfer and diffusion resistance, facilitating pollutant diffusion within carbon materials [32,33]. Additionally, the interlaced carbon fiber network structure enhances adsorption capacity and provides abundant PMS-active sites for NBA.

Defects in materials have been demonstrated to enhance catalytic activity by providing abundant active sites for electron transfer during PMS activation, as evidenced in previous studies [34]. Through Raman spectral analysis, the I_D_/I_G_ ratio (D-band: structural defects vs. G-band: graphitic structure) serves as a critical indicator for quantifying defect density and structural disorder in carbon-based materials [35]. Comparative analysis revealed progressively increasing I_D_/I_G_ values of 0.93 (PB), 0.99 (BC), and 1.16 (NBA), confirming NBA’s superior defect density relative to PB and BC counterparts (Figure 3b). Abundant structural defects may promote the catalytic performance of NBA. The introduction of nitrogen precursors during biomass pyrolysis is conducive to increasing defects, resulting in NBA having the most defective structures. Many defects can alter electron transport pathways and provide sufficient active sites, thereby promoting rapid electron transfer and facilitating PMS activation.

### 3.2. Catalytic Performance Evaluation

**Figure 4:**
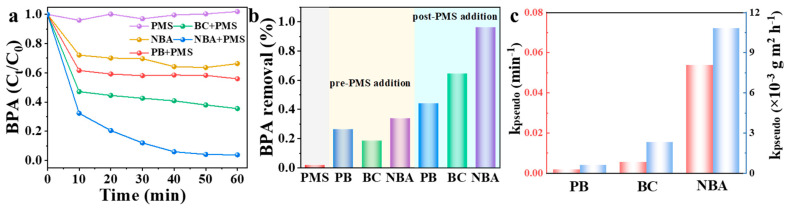
(**a**) Kinetics of BPA removal, (**b**) BPA removal rate, and (**c**) the apparent rate constant (red: k_pseudo_, blue: normalized k_pseudo_) for different catalysts. Conditions: [catalyst] = 0.3 g/L, [PMS] = 1 mM, [BPA] = 10 mg/L, without pH adjustment.

The catalytic efficiencies of various PMS activators were assessed by measuring BPA degradation rates. As shown in Figure 4a,b, BPA degradation was negligible in the PMS-alone system, with only 2% removal within 60 min, indicating that unactivated PMS cannot efficiently oxidize BPA. In the NBA, BC, and PB systems, the BPA degradation efficiencies reached 97%, 64%, and 44% within 60 min, respectively. The activation of PMS and BPA degradation by these three catalysts all followed pseudo-first-order reaction kinetics (Equation (1)). The specific surface area-normalized apparent rate constant (k_pseudo_/S_BET_) of PB, BC, and NBA were 0.6, 2.3, and 10.8 × 10^−3^ g m^−2^ h^−1^, respectively (Figure 4c and Figure S1). Furthermore, a comparative analysis of NBA’s catalytic efficiency in PMS activation with various catalysts (Table S1) revealed that NBA demonstrated reactivity comparable to, or even exceeding, that of high-performance catalysts previously documented. This finding underscores NBA’s exceptional catalytic capability for PMS activation processes and its efficacy in BPA degradation. Notably, PMS (1 mM) alone exhibited minimal BPA degradation, suggesting that BPA cannot be effectively oxidized by PMS without a catalyst. When NBA was directly added without PMS, approximately 34% of BPA was removed from the solution, indicating that NBA has a certain adsorption capacity for BPA. Since PMS activation occurs on the catalyst surface, the adsorption and catalytic capabilities of the catalyst synergistically enhance degradation. The effective adsorption of BPA by NBA reduces the time required for target organic molecules to migrate to its surface and hierarchical pore structure, thereby facilitating attacks by reactive species [36].

ln(C_0_/C_t_) = k_pseudo_ t (1)

where C_0_ and C_t_ are the concentrations of the dyes at time 0 and t, respectively, k = pseudo-first-order rate constant, and t = time in min.

**Figure 5:**
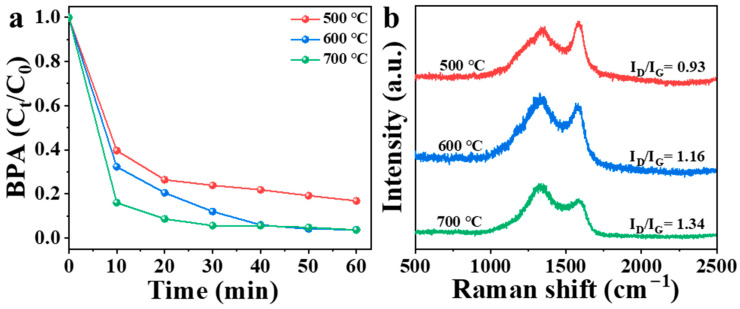
(**a**) Influence of calcination temperature on NBA catalytic activity, (**b**) Raman spectra of materials at different calcination temperatures. Conditions: [catalyst] = 0.3 g/L, [PMS] = 1 mM, [BPA] = 10 mg/L, without pH adjustment.

According to previous studies on biochar, the catalytic performance is affected by calcination temperature [23]. Therefore, three different NBA samples were obtained by calcination at different temperatures in this study (Figure 5a). In the presence of PMS, 83% of BPA was removed by materials calcined at 500 °C within 60 min. Materials calcined at 600 °C and 700 °C showed removal rates of 68% and 84%, respectively, within 10 min, but both reached 97% within 60 min. Increasing the calcination temperature from 500 °C to 600 °C significantly improved the catalytic performance of NBA; however, further increasing the temperature to 700 °C did not enhance its ability to remove organic pollutants substantially. Temperature can regulate the structure, electrical conductivity, oxygen-containing functional groups, and nitrogen speciation in carbon materials [37]. As shown in Figure 5b, the I_D_/I_G_ ratio of NBA gradually increases with rising calcination temperature due to chemical bond cleavage and carbon skeleton reconstruction [38]. Elevated temperatures promote defect site formation and nitrogen species modulation, thereby enhancing NBA’s catalytic activity [39]. However, excessive temperatures may induce carbon–nitrogen bond rupture and heteroatom loss, potentially degrading catalytic performance [37]. Additionally, higher calcination temperatures entail greater energy consumption. Consequently, materials calcined at 600 °C were selected for subsequent experiments. The experimental findings indicated that the NBA/PMS system exhibited enhanced versatility in eliminating diverse electron-rich contaminant species through degradation processes.

### 3.3. Possible Activation Mechanism

**Figure 6:**
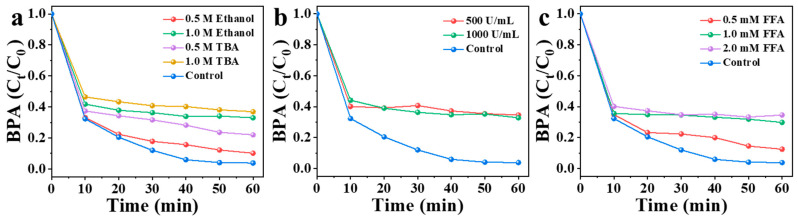
(**a**) Influence of TBA and ethanol as radical scavengers for BPA degradation in NBA/PMS system. (**b**) Influence of SOD as radical scavengers for BPA degradation in NBA/PMS system. (**c**) Influence of different dosages of FFA for BPA degradation in NBA/PMS system. Conditions: [catalyst] = 0.3 g/L, [PMS] = 1 mM, [BPA] = 10 mg/L, without pH adjustment.

The activation of PMS by NBA may degrade BPA through both free radical and non-free radical pathways. To identify the main active species, quenching experiments were conducted. Ethanol, tert-butanol (TBA), furfuryl alcohol (FFA), and superoxide dismutase (SOD) were employed as quenching agents to investigate the mechanism of pollutant degradation by PMS (Figure 6). Specifically, TBA and ethanol were used to probe the involvement of ^•^OH and SO_4_^•−^. Ethanol can quench hydroxyl radicals (^•^OH, K = 1.2–2.8 × 10^9^ m^−1^ s^−1^) and sulfate radicals (SO_4_^•−^, K = 1.6–7.7 × 10^7^ m^−1^ s^−1^), while TBA primarily scavenges ^•^OH (K = 3.8–7.6 × 10^8^ m^−1^ s^−1^) [40]. As shown in Figure 6a, in the NBA/PMS/BPA system, the addition of 0.5 M and 1.0 M ethanol reduced the BPA degradation rate to 90% and 67% within 60 min, respectively. Similarly, 0.5 M and 1.0 M TBA decreased the removal efficiency to 78% and 63%. The stronger inhibitory effect of TBA compared to ethanol may be attributed to its high viscosity masking the active sites on the NBA surface [41].

In addition, SOD and FFA were used as quenchers of superoxide anion radical (O_2_^•−^) and ^1^O_2_, respectively [42]. As illustrated in Figure 6b, the addition of SOD exhibited a significant quenching effect on BPA degradation. Specifically, the degradation efficiency of BPA was suppressed by 31% at an SOD concentration of 500 U/mL. Further increasing the SOD dosage to 1000 U/mL resulted in a comparable inhibition rate of 30%. These results suggest that SOD-mediated scavenging of O_2_^•−^ contributed to approximately 30% of the overall BPA degradation process. ^1^O_2,_ predominantly generated through non-radical pathways in carbon/PMS systems [14,42,43,44], demonstrated critical involvement in BPA degradation. This conclusion was supported by marked suppression of BPA removal efficiency (87% in 60 min) when adding 0.5 mM furfuryl alcohol (FFA), a selective ^1^O_2_ scavenger with a second-order rate constant of 1.2 × 10^8^ M^−1^s^−1^ (Figure 6c) [42]. In the presence of 0.5 mM FFA, only 87% of BPA was removed within 60 min. As the dosage of FFA was increased from 0.5 mM to 1 mM and further to 2 mM, the inhibition rate of BPA degradation efficiency remained nearly constant at ~35%, with no significant enhancement observed (Figure 6c). This plateau suggests that the contribution of singlet oxygen to BPA degradation was approximately 33%. In conclusion, multiple active species, such as ^•^OH, SO_4_^•−^, O_2_^•−^, and ^1^O_2_, were produced in the degradation of BPA by the NBA/PMS system.

**Figure 7:**
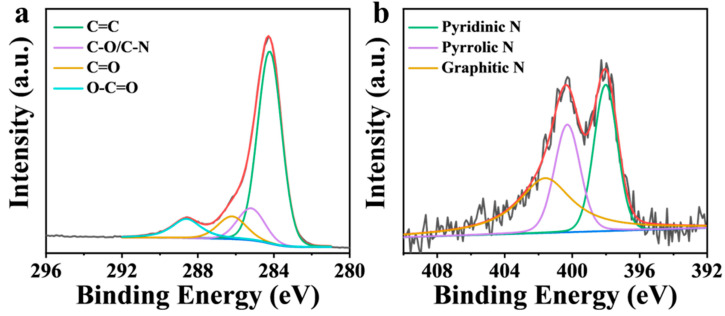
XPS spectra of (**a**) NBA: C1s and (**b**) N1s.

The chemical states of C and N in NBA were analyzed by X-ray photoelectron spectroscopy (XPS). As shown in Figure 7a, the C 1s spectrum of NBA was deconvoluted into four peaks at 284.3 eV (C=C), 285.2 eV (C–O/C–N), 286.2 eV (C=O), and 288.6 eV (O–C=O) [35]. Among them, the percentage of C=O was 9.87%. In the activated PMS system, the presence of C=O contributes both to free radical oxidation (C=O acts as a Lewis base with lone pair electrons, supplying electrons to PMS to split the O–O bond, generating ^•^OH and SO_4_^•−^) and to non-free radical oxidation (^1^O_2_ is produced via the formation of carbonyl-containing intermediates) [45,46,47]. At the same time, the existence of nitrogen species in NBA was analyzed (Figure 7b), and the N 1s via the formation of carbonyl-containing intermediates three peaks at 398.0 eV (pyridine N), 400.3 eV (pyrrole N), and 401.6 eV (graphite N) [35]. The proportions of pyridine N, pyrrolic N, and graphitic N in N species were 32.4%, 27.1%, and 40.5%, respectively. N doping can alter the properties of carbon by modifying the electron distribution and spin density, thereby improving the catalytic performance of carbon materials. Pyridine N and pyrrolic N, as Lewis bases with lone pair electrons, can directly activate PMS to generate ^•^OH and SO_4_^•−^, which are beneficial to the free radical pathway [45]. In contrast, graphitic N tends to induce non-free radical pathways by forming positively charged carbon regions due to the electronegativity difference between N and C atoms [48]. The graphitic N exhibits higher charge density and electron density compared to other N configurations, enabling it to induce electron transfer from adjacent carbon atoms to nitrogen atoms, resulting in positively charged carbon atoms. Simultaneously, it enhances electron transfer between NBA and PMS, forming a metastable intermediate of NBA-PMS. This intermediate can decompose to produce ^1^O_2_ [5].

**Figure 8:**
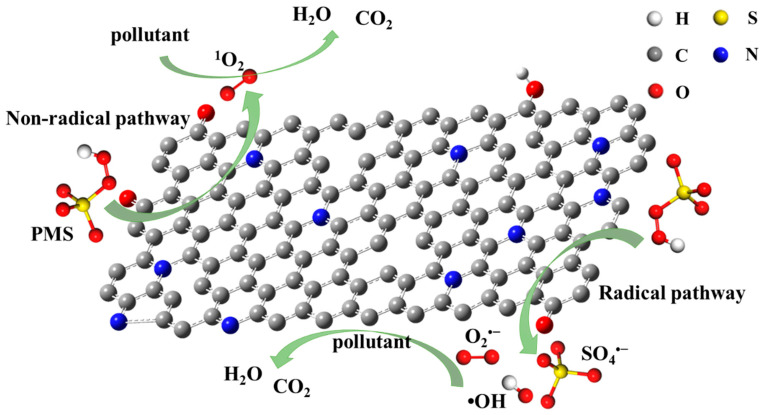
Degradation mechanism of BPA by activation of PMS by NBA.

Due to the high specific surface area and hierarchical porous structure of NBA, the mass transfer and diffusion resistance of NBA are low, and the mass transfer rate is fast. This enables reactant molecules to be more easily adsorbed on their inner surface. In addition, the good adsorption capacity of NBA allows BPA to be enriched on its surface. With the addition of PMS, both free radical and non-free radical pathways are induced. Previous reports have confirmed that the catalytic activity of carbonaceous materials in heterogeneous systems is related to the complex electronic states of covalent carbon systems. Oxygen-containing functional groups and defect sites are the main active sites of carbonaceous catalysts [48,49]. In summary, Lewis bases possessing lone pair electrons, such as pyridine N, pyrrole N, and C=O, are conducive to the activation of PMS to generate free radicals during redox reactions. The non-free radical pathway operates by forming a surface complex between NBA and PMS to facilitate pollutant degradation. Multiple possible active sites exist on NBA, and the activation process is interdependent (Figure 8).

### 3.4. Influence of Operating Parameters on BPA Degradation

**Figure 9:**
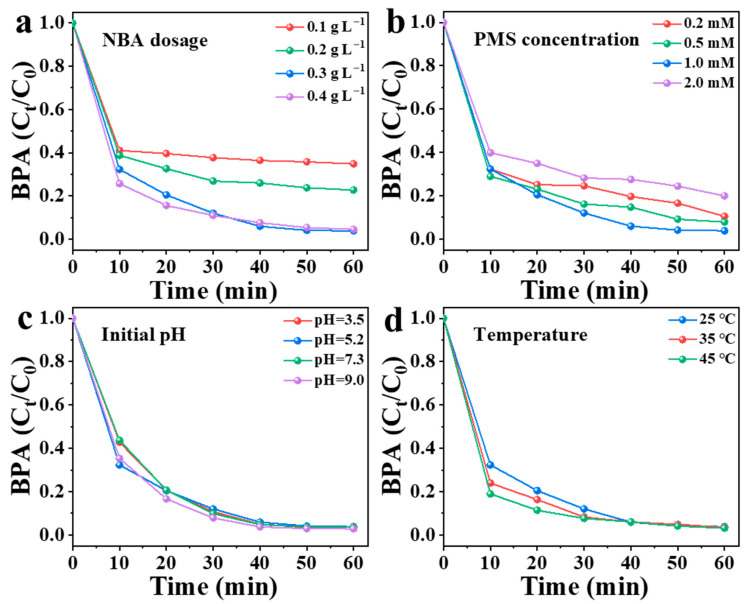
Influence of different conditions in NBA/PMS system: (**a**) NBA dosage, (**b**) PMS concentration, (**c**) initial pH, and (**d**) temperature.

Key operational variables governing BPA degradation in the NBA/PMS system, including NBA dosage, PMS concentration, initial pH, and reaction temperature, were methodically examined through controlled parametric analysis. As shown in Figure 9, when the NBA dosage was increased from 0.1 g/L to 0.3 g/L, the degradation efficiency of BPA improved from 65% to 97%. The removal of BPA by the NBA/PMS system was significantly dependent on the NBA dosage. This is because a higher NBA dosage increases the number of active sites, thereby generating more reactive species [10]. However, when the NBA dosage was further raised to 0.4 g/L, only 95% of BPA was removed within 60 min, with no further improvement in the removal rate. This phenomenon may occur because excessive NBA dosage leads to an overproduction of active species, resulting in radical quenching reactions that interfere with the interaction between reactive species and pollutants (Equations (2)–(4)).

HSO_5_^−^ + ^•^OH → SO_5_^•−^ + H_2_O (2)

HSO_5_^−^ + SO_4_^•−^→SO_5_^•−^ + HSO_4_^−^ (3)

SO_4_^•−^ + SO_4_^•−^ → S_2_O_8_^2−^ (4)

As shown in Figure 9b, PMS concentration was identified as the predominant factor influencing BPA degradation. The degradation efficiency of NBA increased by 7% when the PMS concentration was adjusted from 0.2 mM to 1 mM. This enhancement can be attributed to the activation of more PMS by NBA at higher concentrations, which generated additional active species for pollutant degradation. The BPA removal rate reached its maximum (specific value if available) at 1 mM PMS. However, further increasing the PMS concentration to 2 mM resulted in only an 80% removal efficiency within 60 min, indicating no significant improvement. This phenomenon is likely due to the quenching of excess free radicals under high PMS conditions [18], which reduces the availability of active species for pollutant degradation and may even inhibit the reaction. Therefore, maintaining PMS concentrations within an optimal range (e.g., 1 mM) is critical to minimizing these adverse effects.

When the pH is between 3 and 9, PMS exists in the form of HSO_5_^−^ in the aqueous solution [50]. Figure 9c depicts that the NBA/PMS system can effectively remove BPA in a broad pH range of 3–9. Meanwhile, there was no significant change in solution pH during BPA degradation. The pH range of actual wastewater (5–9) [31] aligns with the operational feasibility of the NBA/PMS system, demonstrating its practicality for real wastewater treatment. As illustrated in Figure 9d, temperature-dependent oxidation kinetics analysis reveals enhanced BPA degradation efficiency in the NBA/PMS system with increasing temperature (25–45 °C). The increase in temperature improved the degradation efficiency of BPA. The reason may be twofold: (1) PMS activation is endothermic, and elevated temperature facilitates the rupture of the O–O bond in PMS [44]; (2) higher temperature accelerates the reaction between reactive species and pollutants, thereby enhancing the reaction rate.

### 3.5. Reusability and Regeneration Performance of NBA

**Figure 10:**
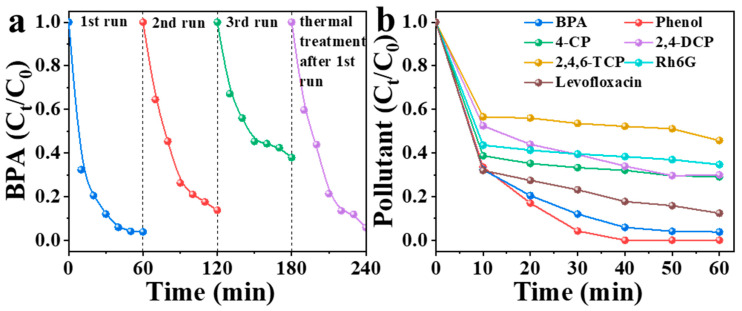
(**a**) Reusability of NBA and (**b**) removal capacity of NBA for different pollutants. Conditions: [catalyst] = 0.3 g/L, [PMS] = 1 mM, [BPA] = 10 mg/L, without pH adjustment.

Catalyst reusability represents a critical performance indicator for assessing industrial viability. To explore the reusability of NBA, four consecutive degradation experiments were conducted (Figure 10a). In the first cycle, NBA achieved 97% BPA removal within 60 min. The removal rates decreased to 86% and 62% in the second and third cycles, respectively. Thermal treatment has been recognized as an effective approach to restoring the catalytic activity of carbon materials [51]. In this study, NBA was regenerated through thermal treatment at 500 °C under a nitrogen atmosphere. Subsequent evaluation assessed the catalytic activity of thermally regenerated catalysts. Results demonstrated that the regenerated NBA regained its activity, achieving 94% BPA removal within 60 min. These findings indicate that NBA can serve as a novel green and sustainable material for wastewater treatment. Furthermore, to assess the universality of NBA, its efficiency in removing diverse organic pollutants was investigated at a concentration of 0.05 mM (Figure 10b). In the NBA/PMS system, the 60 min removal rates for BPA, phenol, 4-CP, 2,4-DCP, 2,4,6-TCP, Rh6G, and levofloxacin were 97%, 100%, 71%, 70%, 54%, 65%, and 88%, respectively. These results confirm that the NBA/PMS system exhibits outstanding catalytic performance for degrading various organic contaminants, positioning it as a promising strategy for pollutant removal.

## 4. Conclusions

In this study, nitrogen-doped biochar aerogel (NBA) was prepared from poplar powder as a biomass source. Under conditions of 0.3 g/L NBA and 1 mM PMS, the NBA/PMS system exhibited excellent degradation efficiency toward diverse pollutants (10 mg/L each), achieving 60 min removal rates of 97% (BPA), 100% (phenol), 71% (4-CP), 70% (2,4-DCP), 54% (2,4,6-TCP), 65% (Rh6G), and 88% (levofloxacin). Moreover, the system showed a good removal rate over a wide pH range. Quenching experiments demonstrated that the degradation of BPA proceeds through radical and non-radical pathways dominated by ^•^OH, SO_4_^•−^, O_2_^•−^ and ^1^O_2_. XPS analysis was also used to further investigate the possible active sites of NBA in activating PMS. The NBA/PMS system exhibits good recyclability and broad applicability to various pollutants, suggesting its potential for practical wastewater treatment.

## Data Availability

The data that support the findings of this study are available from the corresponding author upon reasonable request.

## Supplementary Materials

The following supporting information can be downloaded at: https://www.mdpi.com/article/10.3390/nano15110865/s1, Figure S1: Kinetics of BPA degradation in the different systems; Table S1: Comparison of catalytic performance of NBA with other catalysts on PMS/PS activation for BPA degradation [52,53,54,55,56,57,58,59,60,61,62,63,64,65].
