# Supplementary material for: Nitrogen-Doped Biochar Aerogel as Efficient Peroxymonosulfate Activator for Organic Pollutant Removal
Source: Nanomaterials (Basel). 2025 Jun 4;15(11):865. doi: 10.3390/nano15110865 (PMC12158100; doi:10.3390/nano15110865)
Supplement: Supplementary file 1 [file nanomaterials-15-00865-s001.zip › nanomaterials-3645790-supplementary.pdf]

nanomaterials

Nitrogen-Doped Biochar Aerogel as Efficient Peroxymonosulfate Activator for Organic Pollutant Removal

Lingshuai Kong [1]*, Mingshuo Zhu [2,3] and Jinhua Zhan [1,2]*

[1] Institute of Eco-Environmental Forensics, School of Environmental Science and Engineering, Shandong University, Qingdao, 266237 China
[2] Key Laboratory of Colloid and Interface Chemistry, Ministry of Education, School of Chemistry and Chemical Engineering, Shandong University, Jinan, 250100 China
[3] Shandong Coal Science and Technology Research Institute Branch, Yankuang Energy Group Co., Ltd., Jinan, 250014, China
* Correspondence: Correspondence: qfkongls@sdu.edu.cn (L.K.), jhzhan@sdu.edu.cn (J.Z.)

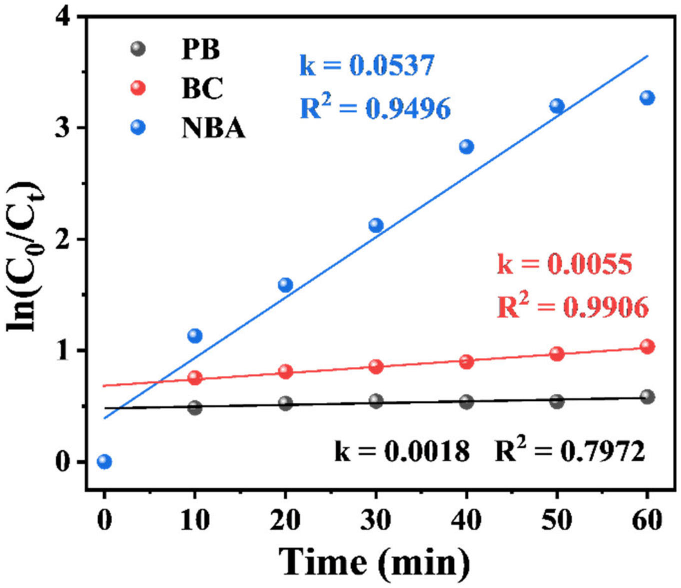

Figure S1. Kinetics of BPA degradation in the different systems. Conditions: [catalyst] = 0.3 g/L, [PMS] = 1 mM, [BPA] = 10 mg/L, without pH adjustment.

Table S1. Comparison of catalytic performance of NBA with other catalysts on PMS/PS activation for BPA degradation.

Catalysts	Experimental conditions	Degradation time (efficiency)	Active mechanism	Ref.
Mn-doped biochar (Mn-TPs-900)	[catalyst] = 0.2 g/L, [phenol] = 50 mg/L, [PMS] = 3.2 mM	60 min (91.2%)	Free radicals, high-valent metals, $^1O_2$, and ETP	[1]
alkali-activated biochar (KBC)	[catalyst] = 0.15 g/L, [HA] = 20 mg/L, [PMS] = 1.0 mM	120 min (98%)	$^1O_2$	[2]
N, S co-doped/biochar (NSBC)	[catalyst] = 0.2 g/L, [Tetracycline] = 20 mg/L, [PMS] = 1.0 mM	40 min (90.02%)	Free radicals, $^1O_2$, and ETP	[3]
$MnFe_2O_4$ modified biochar composites (BC-$FM_{0.25}$)	[catalyst] = 0.5 g/L, [Sulfamethazine] = 50 mg/L, [PMS] = 0.5 g/L	30 min (98.60 %)	$^1O_2$ and high-valent metal	[4]
biochar loaded with both CoN_3 and CuN_3O_2 sites	[catalyst] = 0.02 g/L, [Sulfamethoxazole] = 15 mg/L, [PMS] = 1.2 mM	60 min (93%)	•OH, $SO_4^{\bullet-}$, and $^1O_2$	[5]
Engineered digestate-derived biochar (KNBC750)	[catalyst] = 1 g/L, [oxytetracycline] = 15 mg/L, [PMS] = 5 mM	30 min (100 %)	$SO_4^{\bullet-}$ and $^1O_2$	[6]
Pyrite/biochar	[catalyst] = 0.05 g/L, [Tetracycline] = 20 mg/L, [PMS] = 1.0 mM	60 min (91%)	$^1O_2$	[7]
B, N co-doped biochar	[catalyst] = 0.2 g/L, [AO7] = 50 mg/L, [PMS] = 1.0 mM	40 min (97.4%)	ETP	[8]
natural pyrite-biochar composite	[catalyst] = 1.0 g/L, [Sulfamethoxazole] = 10 mg/L, [PMS] = 0.5 mM	120 min (95.5%)	$^1O_2$	[9]
nitrogen, nickel dual-site biochar	[catalyst] = 0.1 g/L, [norfloxacin] = 30 mg/L, [PMS] = 0.25 mM	100 min (94.43%)	$^1O_2$ and $O_2^{\bullet-}$	[10]
Porphyra-derived porous biochar	[catalyst] = 0.4 g/L, [carbamazepine] = 20 mg/L, [PMS] = 0.4 g/L	40 min (92%)	Free radicals, $^1O_2$, and ETP	[11]
nitrogen-doped biochar (N-C-d-4-800)	[catalytic] = 0.5 g/L, [BPA] = 10 mg/L, [PMS] = 2.0 mM	5 min (100%)	Free radicals and $^1O_2$	[12]
N-doped biochar (NSOBC-6)	[catalytic] = 0.3 g/L, [naproxen] = 20 mg/L, [PMS] = 0.2 g/L	60 min (100%)	$^1O_2$ and $O_2^{\bullet-}$	[13]
highly nitrogen-doped biochar	[catalytic] = 1.0 g/L, [RB5] = 125 mg/L, [PMS] = 5.0 mM	60 min (100%)	$^1O_2$	[14]
nitrogen-doped biochar aerogel (NBA)	[catalytic] = 0.3 g/L, [BPA] = 10 mg/L, [PMS] = 1.0 mM	60 min (97%)	Free radicals and $^1O_2$	this work

References

1. Meng, H.; Zhou, J.; Nie, C.; Li, W.; Li, D.; Zhang, Y.; Ao, Z. Insights into Mn-doped biochar induce peroxymonosulfate activation for phenol degradation: The overlooked significance of C-O-Mn. *Journal of Hazardous Materials* **2025**, *492*, 138031, doi:https://doi.org/10.1016/j.jhazmat.2025.138031.
2. Wang, H.; Qiao, C.; Chen, C.; Liu, B.; Du, J.; Wu, Q.; Feng, X.; Zhan, S.; Guo, W.-Q. Synergistic adsorption and singlet oxygen-ation of humic acid on alkali-activated biochar via peroxymonosulfate activation. *Chinese Chemical Letters* **2025**, *36*, 110244, doi:https://doi.org/10.1016/j.cclet.2024.110244.
3. Tang, F.; Dai, H.; Yang, X.; Li, W.; Wang, B. Nitrogen and sulfur co-doped watermelon rind as an ordered mesoporous biochar activated peroxymonosulfate (PMS) for efficient tetracycline degradation. *Journal of Environmental Chemical Engineering* **2024**, *12*, 112302, doi:https://doi.org/10.1016/j.jece.2024.112302.
4. Chen, L.; Wang, X.; Yuan, M.; Ni, B.-J.; Xia, S.; Zhao, J. Insights into the removal of sulfamethazine and sulfonamide-resistant bacteria from wastewater by Fe-Mn spinel oxide modified cow manure biochar activated peroxymonosulfate: A nonradical pathway regulated by enhanced adsorption and 3d orbital electron reconstruction. *Applied Catalysis B: Environment and Energy* **2025**, *361*, 124652, doi:https://doi.org/10.1016/j.apcatb.2024.124652.

5. Wang, C.; Tian, J.; Cui, Y.; Li, N.; Cui, X.; Yan, B.; Chen, G. Synergy of CoN_3 and CuN_3O_2 sites in single atom-decorated biochar for peroxymonosulfate activation: Accelerating the production of $SO_4^{\bullet -}$ and •OH. *Chemical Engineering Journal* **2024**, *496*, 154133, doi:https://doi.org/10.1016/j.cej.2024.154133.
6. Akaniro, I.R.; Zhang, R.; Chai, X.; Tsang, C.H.M.; Wang, P.; He, S.; Yang, Z.; Zhao, J. Engineered digestate-derived biochar mediated peroxymonosulfate activation for oxytetracycline removal in sustainable wastewater remediation. *Environmental Pollution* **2024**, *360*, 124640, doi:https://doi.org/10.1016/j.envpol.2024.124640.
7. Fang, J.; He, F.; Yan, Z.; Wang, J.; Yu, R.; Zhou, H. Pyrite/biochar-activated peroxymonosulfate strengthens tetracycline degradation: Important roles of surface functional groups and Fe(II)/Fe(III) redox cycling. *Journal of Environmental Chemical Engineering* **2024**, *12*, 112923, doi:https://doi.org/10.1016/j.jece.2024.112923.
8. Pan, M.; He, Z.; Yang, X. Functional biochar accelerates peroxymonosulfate activation for organic contaminant degradation via the specific B–C–N configuration. *Chemosphere* **2024**, *365*, 143202, doi:https://doi.org/10.1016/j.chemosphere.2024.143202.
9. Zhao, R.; Wang, T.; Wang, Z.; Cheng, W.; Li, L.; Wang, Y.; Xie, X. Activation of peroxymonosulfate with natural pyrite-biochar composite for sulfamethoxazole degradation in soil: Organic matter effects and free radical conversion. *Journal of Hazardous Materials* **2024**, *469*, 133895, doi:https://doi.org/10.1016/j.jhazmat.2024.133895.
10. Cheng, L.; Lu, H.; Xu, C.; Meng, J.; Luo, J.; Jiang, J.; Qin, H. The efficient degradation of high concentration norfloxacin by nitrogen, nickel dual-site biochar activated peroxymonosulfate: Performance and mechanism. *Journal of Environmental Chemical Engineering* **2025**, *13*, 116950, doi:https://doi.org/10.1016/j.jece.2025.116950.
11. Peng, X.; Li, Y.; Jiang, Z.; Zhu, K.; An, Q.; Xiao, Z.; Dong, X.; Zhai, S. Photothermal-synergistic peroxymonosulfate activation promoting carbamazepine degradation by Porphyra-derived porous biochar composites: Performance, mechanism, transformation pathway and practical application. *Chemical Engineering Journal* **2024**, *489*, 151263, doi:https://doi.org/10.1016/j.cej.2024.151263.
12. Xu, L.; Wu, C.; Liu, P.; Bai, X.; Du, X.; Jin, P.; Yang, L.; Jin, X.; Shi, X.; Wang, Y. Peroxymonosulfate activation by nitrogen-doped biochar from sawdust for the efficient degradation of organic pollutants. *Chemical Engineering Journal* **2020**, *387*, 124065, doi:https://doi.org/10.1016/j.cej.2020.124065.
13. Zhang, Y.; Li, M.; Shi, Y.; Zhang, H.; Deng, H.; Xia, D. Efficient activation of peroxymonosulfate by N-doped waste herb senna obtusifolia biochar for degrading NPX: Synergistic effect of carbonyl and nitrogen sites. *Journal of Environmental Management* **2024**, *371*, 123207, doi:https://doi.org/10.1016/j.jenvman.2024.123207.
14. Zhu, Z.; Yang, X.; Ye, X.; Li, Q.; Wang, J.; Wu, L.; Huang, Z.-H.; Wang, M.-X. Activating peroxymonosulfate by high nitrogen-doped biochar from lotus pollen for efficient degradation of organic pollutants from water: Performance, kinetics and mechanism investigation. *Separation and Purification Technology* **2024**, *346*, 127456, doi:https://doi.org/10.1016/j.seppur.2024.127456.
